# Seroprevalence of Fowl Pox Antibody in Indigenous Chickens in Jos North and South Council Areas of Plateau State, Nigeria: Implication for Vector Vaccine

**DOI:** 10.5402/2012/154971

**Published:** 2012-09-25

**Authors:** Meseko Clement Adebajo, Shittu Ismail Ademola, Akinyede Oluwaseun

**Affiliations:** ^1^Viral Research Department, National Veterinary Research Institute, Vom Nigeria, Nigeria; ^2^Department of Medicine, The John Hopkins University School of Medicine, Baltimore, MD, USA

## Abstract

Fowl pox is a viral disease of domestic and wild birds. The large size of the genome makes it a useful vector for recombinant DNA technology. Although the disease has been described in both commercial and indigenous chickens in Nigeria, data are limited on seroprevalence in free range chickens. Such data are, however, important in the design and implementation of fowl pox virus vector vaccine. We surveyed current antibody status to fowl pox virus in free range chickens by testing 229 sera collected from 10 villages in Jos North and Jos South LGA of Plateau State Nigeria. Sera were analyzed by AGID against standard fowl pox antigen. Fifty-two of the 229 (23%) tested sera were positive for fowl pox virus antibody, and the log titre in all positive specimen was >2. Thirty (21%) and twenty-two (27%) of the samples from Jos South and Jos North, respectively, tested positive. This was, however, not statistically significant (*P* = 0.30). Generally the study showed a significant level of antibody to fowl pox virus in the study area. This observation may hinder effective use of fowl pox vectored viral vaccine. Fowl pox control is recommended to reduce natural burden of the disease.

## 1. Introduction

Fowl pox is a viral disease caused by avipoxvirus belonging to the chordopoxvirinae subfamily of the poxviridae family, which induces pustular, benign, and proliferative lesions of the skin and diphtheritic lesions on the mucous membrane of the digestive and respiratory passages [1, 2]. The disease affects both domestic and free living birds in nature resulting in varying morbidity and mortality [3]. The diphtheritic form is usually more severe as it causes significant mortality and economic losses in affected flocks [4]. Although fowl pox is believed to be widespread in backyard and to some extent intensively reared poultry flocks in Nigeria [5], the epidemiologic details of the disease are not quite clear in free range indigenous chickens. While the virus is transmitted mechanically through wounds on the skin, biting insects such as mosquitoes and mites are also common vectors [6, 7]. Aerosols generated from infected birds or ingestion of contaminated food or water has also served as source of transmission [8], hence birds on free range may be clinically or subclinically infected and develop antibody to fowl pox virus through many of these ubiquitous exposure. 

**Figure 1:**
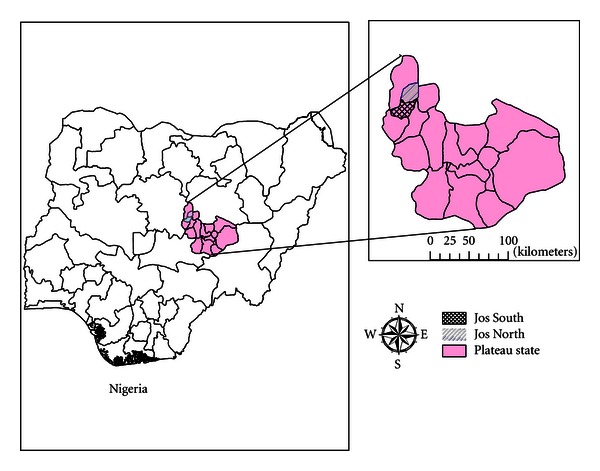
Map of Nigeria, showing Plateau state and Jos North and South Local Government Areas.

The recent advance in vaccinology takes advantage of subunit of pathogens and delivery of multivaccine candidates [9]. The large size genome (200 kbp) of the fowl pox virus is used in recombinant DNA technology to insert genes of interest that may be delivered to recipient host as vector vaccine. However, absence of or low level antibodies to fowl pox virus are requirements for effective immunization with fowl pox vector vaccine in infectious disease control. This study evaluates the current natural antibody profile to fowl pox virus in free range indigenous chickens in villages in and around Jos in Plateau State, Nigeria (Figure 1).

## 2. Material and Method

To detect fowl pox antibody in unvaccinated indigenous chickens on free range, two hundred and twenty-nine conveniently sampled birds were bled by vein puncture in 10 villages in Jos North and South LGA of Plateau State as shown in Table 1. Sera obtained were tested by Agar Gel Immuno-diffusion (AGID) against standard fowl pox antigen and antiserum (Charles River Laboratory, USA), according to OIE protocols [10]. The gel-diffusion medium was prepared with 1% agar and 8% sodium chloride in distilled water. Precipitating antibodies were detected by reacting test sera against viral antigens placed in central wells of agar gel and test sera in the peripheral wells. Positive and negative control sera were included as internal controls. The plates were thereafter incubated at 25°C room temperature and after 24–48 hours of incubation, precipitation lines were observed between homologous antibody and antigen indicating positive results. Positive samples were titrated by making twofold serial dilutions and tested again by AGID as described earlier. The proportions of positive and negative samples were compared using chi-squared tests.

## 3. Results and Discussion

Fifty-two of the 229 (23%) of tested sera showed line of precipitation similar to positive controls and were taken as positive for fowl pox antibody. The titre in all cases was >2log_2_. Thirty (21%) of the 146 samples from Jos South and twenty-two (27%) of the 83 samples from Jos North tested positive. In Jos South council area, the village with the highest number of positive samples was Du, where 29% of the samples tested positive. The village with the lowest proportion of positive samples in Jos South was Rahol Kanang, which had 14% seroprevalence. There was, however no statistically significant difference in seroprevalence among the villages (*P* = 0.67). In Jos North, Naraguta had the highest seroprevalence of 35%, closely followed by Yan Trailer, which had 33% seroprevalence. The village with the lowest seroprevalence in Jos North was Angwan Soya (17%); these differences in seroprevalence were also not statistically insignificant (*P* = 0.51). Overall, Jos North had a higher seroprevalence of 27% over Jos South (21%). However, this difference was also not statistically significant (*P* = 0.30). 

 Overall seroprevalence of 23% fowl pox antibody level in indigenous chicken in North Central Nigeria using AGID test is less than 89% observed by Ohore et al., [11] in unvaccinated indigenous chickens using ELISA technique in the South West region of Nigeria. A similar work in Zaria (North West Nigeria) by Saidu et al., [12] using AGID, however, indicated 5% seroprevalence which is much lower than our observation. The consistency of antibody detection in local chickens with previous studies indicates preponderance of significant antibody to fowl pox among indigenous chickens in the study areas. Though conventional serological techniques of passive neutralization and agar-gel immunodiffusion are still globally used for surveillance and diagnosis in poultry [12–14], sensitivity of AGID appears to be low when compared with other detection method such as enzyme-linked immunosorbent assay—ELISA [11, 15]. ELISA is a nonspecies specific test for birds [16], it is also faster and easier method to detect antibodies against fowl pox, particularly when large numbers of sera are involved, though it is not as specific as AGID [17]. ELISA protocols have also been developed and used to test the efficacy of fowl pox vaccines in commercial and wild birds where AGID is ineffective due to lack of precipitating antibodies [18, 19]. However, in species like chicken with precipitating antibody to fowl pox, AGID is still a useful test because of its simplicity in terms of test reagents, equipment and analysis that can readily be performed in standard laboratories with low budget. The high sensitivity and less specificity of ELISA also make it prone to false positive results [16]. This may account for 89% antibody level to fowl pox virus reported by Ohore [11] and coworkers in local chickens. A parallel test using AGID and ELISA may provide better understanding.

In the control of avian influenza and other infectious diseases in poultry, depopulation of infected flocks in combination with vaccination of population at risk is considered more effective [20, 21]. Recombinant fowl pox virus vaccine carrying avian influenza virus H5 haemagglutinin (HA) has been used with varying degree of success [21]. This may not be unconnected with previous exposure of chickens to fowl pox virus, which can be responsible for inconsistency in protection for birds immunized with the fowl pox virus-vectored vaccine [22]. In this study, we detected significant levels of antibody to fowl pox in free range indigenous chickens; this is likely due to natural exposure as there were no indication that the birds were vaccinated. This observation may hinder effective use of fowl pox vector viral vaccine in local birds, which are considered population at risk because of their frequent contact with wild or feral birds that may asymptomatically harbor viral diseases like influenza [23]. Similarly, attempt to use fowl pox vector vaccine in commercial flocks with residual antibodies from vaccination may also be counterproductive. Control of fowl pox is thus recommended to reduce the burden of disease and promote efficiency of future immunization to fowl pox recombinant DNA subunit or vector vaccines.

## Figures and Tables

**Table 1:** Distribution of samples collected from Jos South and Jos North LGA of plateau state.

Villages	LGA	No. of samples collected	No. positive	Percentage of positive samples	Titres
Jos South					
Du	Jos South	28	8	29	2^2^
Foron	Jos South	19	5	26	2^3^
Vwang	Jos South	25	6	24	2^2^
Rantya	Jos South	15	2	13	2^2^
Rahol Kanang	Jos South	35	5	14	2^2^
Shaka	Jos South	24	4	17	2^2^

Total		146	30	21	

Jos North					
Naraguta	Jos North	20	7	35	2^2^
Yan trailer	Jos North	18	6	33	2^2^
Angwa soya	Jos North	18	3	21	2^3^
Rukuba	Jos North	27	6	22	2^2^

Total		83	22	27	

^∗^Percentages rounded up to nearest whole number.
